# The Biopsychosocial Burden of Prostate Biopsy at the Time of Its Indication, Procedure, and Pathological Report

**DOI:** 10.1155/2019/2653708

**Published:** 2019-04-01

**Authors:** Walker W. Laranja, Brunno C. F. Sanches, Brunno R. I. Voris, João C. C. Alonso, Fabiano A. Simões, Ronald F. Rejowski, Leonardo O. Reis

**Affiliations:** ^1^University of Campinas (Unicamp), Campinas, São Paulo, Brazil; ^2^Pontifical Catholic University of Campinas (PUC-Campinas), Campinas, São Paulo, Brazil; ^3^Paulínia Municipal Hospital (HMP), Paulínia, SP, Brazil

## Abstract

**Purpose:**

To explore the burden of prostate biopsy at the time of its indication, procedure, and pathological report in the prostate cancer-screening scenario that is neglected and underestimated in the literature.

**Methods:**

Prostate biopsy was offered to 47 consecutive patients with prostate-specific antigen (PSA) over 4 ng/dl or suspicious digital rectal examination (DRE) of whom 16 had undergone a biopsy. Comprehensive validated questionnaires at Time 0 (prebiopsy), Time 1 (before diagnosis, 20 days after biopsy), and Time 2 (after diagnosis, 40 days after biopsy) accessed patients' erectile (IIEF-5) and voiding (IPSS) functions, Beck scales measured anxiety (BAI), hopelessness (BHS), and depression (BDI), added to the emotional thermometers including five visual analog scales for distress, anxiety, depression, anger, and need for help. The Mann-Whitney or Friedman tests were obtained among times and studied variables.

**Results:**

Prostate biopsy did not significantly impact patients' erectile and voiding functions while a higher Beck anxiety index (BAI) was observed at Time 0 (6.89 ± 6.33) compared to Time 1 (4.83 ± 2.87),* p*=0.0214, and to Time 2 (4.22 ± 4.98),* p*=0.0178. At Time 0, patients that experienced a previous biopsy presented higher distress (3.1 ± 3.0 vs. 1.6 ± 2.3),* p*=0.043, and emotional suffering thermometer scores (2.3 ± 3.3 vs. 0.9 ± 2.4) compared to those undergoing the first biopsy,* p*=0.036. At Time 2, patients with positive biopsies compared with those with negative ones showed no significant difference in outcome scores. The sample power was >90%.

**Conclusions:**

To be considered in patients' counseling and care, the current study supports the hypothesis that the peak burden of prostate biopsy occurs at the time of its indication and might be higher for those experiencing rebiopsy, significantly impacting patients' psychosocial domains.

**Trial Approval:**

This trial is registered under number NCT03783741.

## 1. Introduction

Prostate cancer is a very common condition, especially with aging. Enhancement of life expectancy around the world consequently promotes an increase of at least 60% in the diagnosis of prostate cancer. In absolute terms, it is the sixth most common tumor in the world and the most prevalent in men, accounting for about 10% of all cancers [1, 2].

The biopsy is considered the best form of histopathological prostate cancer diagnosis [3]. This diagnosis method may represent a potent psychological stress factor. In extreme cases it can increase the risk of negative impacts on recovery and death from cardiovascular diseases, especially after diagnosis [4–6].

Recognizing the real impact of this procedure in the physical as well as the psychological context is crucial to provide better support to the patient and minimize side effects that can hinder posterior treatment.

The aim of this trial is to explore the burden of prostate biopsy (PBx) at time of its indication, procedure, and pathological report in the prostate cancer-screening scenario that is neglected and underestimated in the literature.

## 2. Methods

This is a prospective, longitudinal, and observational study in which the sexually active patients were evaluated at the Urology Department of the city of Paulínia and submitted to biopsy guided by transrectal ultrasound after ethics committee (355.357) and trial (NCT03783741) approvals.

Consecutive patients attended to by a urologist and with present prostate cancer suspicions (PSA> 4 ng/dL and/or rectal examination, DRE) [7] were submitted for a PBx. They were evaluated at three different moments of the biopsy:

1. Seven days before the biopsy procedure (T0)
2. 20 days after the biopsy, upon receiving the histopathological result, before becoming aware of it (T1)
3. 40 days after the biopsy, 20 days after being aware of the test result (T2)

**Figure 1:**
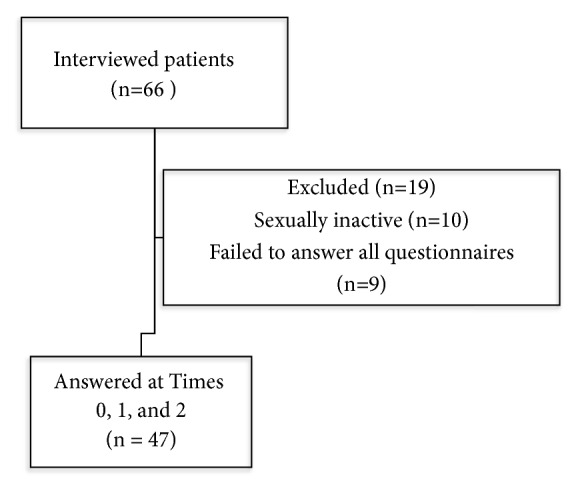
Study flowchart.

Sixty-one consecutive patients were invited to participate in the study; 10 of them had no active sexual life and 9 answered the questionnaires only at the first moment and were excluded; 47 responded at the three times (T0, T1, and T2) (Figure 1).

Validated instruments were applied: IIEF-5 (erectile function); IPSS (voiding function); Beck scales (BAI) (anxiety), BHS (hopelessness), BDI (depression), and emotional thermometers.

The comparison among the moments (T0, T1, and T2) was performed through the Friedman test (analysis of variance) for repeated measures with the variables transformed in stations. The comparison between patients and variables was performed using the Mann-Whitney test [8–10]. Multivariate analysis searched for independent significant variables, the sample power was calculated, and the level of significance considered was 5%.

## 3. Results

Confirmed PCa through biopsies occurred in 32% (n = 15). The ages varied between 40 and 81 years (mean 62.37 ± 7.97), and 34% (n = 16) of the patients had already had at least one previous biopsy performed.

There was no significant impact on erectile and voiding functions throughout the assessments while a higher Beck anxiety index (BAI) was observed at Time 0 (6.89 ± 6.33) compared to Time 1 (4.83 ± 2.87),* p*=0.0214, and to Time 2 (4.22 ± 4.98),* p*=0.0178 (Table 1).

At Time 0, patients that experienced a previous biopsy presented higher distress (3.1 ± 3.0 vs. 1.6 ± 2.3),* p*=0.043, and emotional suffering thermometer scores (2.3 ± 3.3 vs. 0.9 ± 2.4) compared to those undergoing the first biopsy,* p*=0.036 (Table 2).

At Time 2, patients with positive biopsies compared with those with negative ones showed no significant difference in outcome scores. The sample power was >90%.

On multivariate analysis BAI was the only independent variable in the PBx timeline,* p*=0.0312.

## 4. Discussion

In the present study, although PBx does not have a significant short- and medium-term impact on the erectile and voiding functions, there is interference mainly in anxiety (BAI), emotional distress, and distress, significantly higher at the time of biopsy indication, when compared to subsequent times, even in cases of positive biopsy for PCa.

Thus, it is intriguing to note that although we observed greater emotional distress and anxiety before the procedure, these feelings were even greater in the patients submitted to rebiopsy when compared to those that have never had this done before and there was no difference between the positive and negative results for cancer.

These data will allow better patient care in the context of the early diagnosis of PCa, in order to identify, in addition to sensitive aspects, also the critical moment (prebiopsy) to implement psychosocial actions that will minimize the impact of health interventions such as PBx, culminating in a better quality of life.

It is important to note that the scope of the study in question is in line with a current discussion that seeks to replace the biomedical model with a technical-instrumental reference of the biosciences by the biopsychosocial model with a broad and integral view of being and falling ill that includes the physical, psychological, and social aspects [11].

It is necessary to improve the doctor-patient relationship considering understanding the disease. It is important to improve communication, increasing flexibility, treating malaise and disease, respecting diversity, and assessing the patient's previous historical context, aiming to supply the needs of the patient to find ways that will allow converging to the same point aspects between the doctor and the patient in the same context [12].

It is noteworthy that the psychosocial side effects of prostatic biopsy have also attracted more attention recently, especially in relation to emotional issues [13] and although we have found important statistical differences among the cited scores, it is quite complex to define the real clinical impact of interventions such as PBx in the psychosocial context [14].

In the on-screen study we consider the hypothesis that stress-triggering factors and their organic consequences occur differently in three moments:

- T0: conflicts and concerns arise with the biopsy procedure and with its result (chance of cancer diagnosis)
- T1: with the proximity of the results of the histopathological report, concerns were focused on the diagnosis and the chance of cancer; also 20 days after the biopsy, residual organic side effects are rare, as previously described by our group [15]
- T2: the subject is dealing with one of 2 possible scenarios: positive or negative biopsy for prostate cancer

The erectile and voiding functions were not significantly affected when compared the patient's own situations before PBx. In addition, the patients were more anxious before the procedure compared to waiting for the diagnosis; Wade et al. identified that anxiety levels increased 7 days after the biopsy procedure, dropping at 35 days, maintaining an increased anxiety only for patients who had a diagnosis of PCa, suggesting that a broader pre-PBx approach may reduce anxiety after the procedure [13].

In a study developed by Klein et al. [16] analyzing effects 1, 4, and 12 weeks after PBx in the erectile, voiding, and quality-of-life effects, a greater voiding dysfunction was observed in the first and fourth weeks after PBx, which returned to normal levels. Regarding erectile function, there was no significant impact, except for transitory worsening only in patients with the previous dysfunction. Interestingly, even without directly asking about anxiety and not having a prebiopsy evaluation, some patients were anxious after PBx in the study.

Helfand et al. studying LUTS, ED, and quality of life in patients submitted to PBx observed significant alterations only in the erectile function of patients who had confirmed PCa [17], allowing the interpretation of possible emotional interference of biopsy positivity as a cause of ED in these cases.

Zisman et al., who also evaluated pain, anxiety, and erectile function, found 15% of patients with transient ED up to 30 days after PBx, and the highest level of anxiety was detected in waiting for the histopathological result [18], opposing our study in which the greatest anxiety was observed before the biopsy. This fact makes us reflect on the importance of elucidating doubts and presenting statistical data that will make the patient more confident and less anxious about the procedure and its results.

In a study by Chrisofos et al. [19] excluding factors of comorbidities that would lead to ED as in the present study, there was also no significant erectile impact after the biopsy.

The present study, of a prospective, observational, and longitudinal nature, reports the daily practice of a urology health service. Therefore, the extracted data are applicable to the daily clinical practice, since an artificial condition for the research was not prepared. In this context, the mean age was 62 years and the function scores reveal mild erectile dysfunction and voiding symptom in this population.

Although the number of patients was relatively small, and there were patient losses, especially in the last interview, the power of the sample was adequate for the presented results (> 90%) and approximately 50 patients were evaluated before and after the biopsy [20].

The prospective and longitudinal perspective of the study allows a better definition of causes and effects in the studied context, minimizing erroneous conclusions when compared to cross-sectional and retrospective studies.

However, the inclusion of a greater number of subjects and subsequent evaluations after 40 days of biopsy will allow the analysis of subgroups of age extremes, erectile and voiding functions, and long-term data in future studies.

## 5. Conclusion

In the context of the early diagnosis of PCa, there was no significant impact on erectile (IIEF-5) and voiding (IPSS) functions throughout the short- and mid-term evaluations after PBx. However, there were higher anxiety indexes (Beck inventory and emotional thermometers), especially at the time of the indication and in cases with previous biopsy experience, with no difference between patients with a positive and negative result for cancer.

## Figures and Tables

**Table 1:** Scores at Times 0, 1, and 2.

Tools	T0	T1	T2	*p* value	Sample power
IIEF	15.83 ± 8.52	17.67 ± 8.85	18.06 ± 8.05	ns	

IPSS	6.17 ± 5.85	6.22 ± 5.39	4.79 ± 4.81	ns	

BAI	6.89 ± 6.33	4.83 ± 2.87	4.22 ± 4.98	0.0178	0.938

BDI	5.61 ± 8.32	4.33 ± 5.71	5.06 ± 6.72	ns	

BHS	3.12 ± 3.48	1.94 ± 2.41	3.12 ± 3.14	ns	

**Table 2:** Thermometer scores comparison according to previous biopsy at Time 0.

Tools	First biopsy (mean ± SD)	>1 biopsy (mean ± SD)	*p* value
Emotional suffering	0.9 ± 2.4	2.3 ± 3.3	* 0.036*

Distress	1.6 ± 2.3	3.1 ± 3.2	*0.043*

## Data Availability

The data used to support the findings of this study are included in the article.
